# Editorial: Insights in social psychiatry and psychiatric rehabilitation: 2022: Foreword

**DOI:** 10.3389/fpsyt.2024.1378871

**Published:** 2024-03-25

**Authors:** Antonio Vita, Jacopo Lisoni

**Affiliations:** ^1^ School of Medicine, University of Brescia, Brescia, Italy; ^2^ Department of Mental Health- Spedali Civili Hospital, Brescia, Italy

**Keywords:** social psychiatry, psychiatric rehabilitation, severe mental disease, innovation, recovery

This Research Topic presents insightful results on different topics in the field of Social Psychiatry and Psychiatric Rehabilitation, including the role of recovery assessment, the need to promote social/work support, to improve quality of life through recovery-oriented programs, the needs of highly disadvantaged patient population, and the need to expand shared-decision making actions in psychiatry and psychosocial rehabilitation.

Considering advancement in assessing the recovery process, insights come from von Peter et al. and Lakshman et al. The formers presented initial results on the development of a new research tool, the *Needs and Experiences in Psychiatric Treatment (NEPT*), to evaluate the experiences and fulfillment of needs during psychiatric treatment from the perspective of users. This tool was developed during a participatory-collaborative process evaluation from controlled, multi-center, prospective cohort study, aiming at evaluating psychiatric Flexible and Integrative Treatment models. The research team consisted of researchers with and without experiential expertise. As NEPT showed good psychometric properties, NEPT was considered a promising tool for further development to assess the experiences and fulfillment of needs of psychiatric care models from a user’s perspective. Lakshman et al. developed a Malay-language version of the *Recovery Knowledge Inventory*, one of the most influential scales to assess knowledge and attitudes toward recovery-oriented practices among mental health providers. The study demonstrated that, while the original 20-item scale did not exhibit sufficient validity among Malaysian mental health workers, the modified 11-item Malay-version had good construct validity for application in clinical practice.

Then, results from systematic reviews come from Chirio-Espitalier et al. and Cheng and Lai. Considering that Personal Recovery (PR) is an important mental health goal, Chirio-Espitalier et al. conducted a systematic review on 24 papers evaluating PR in bipolar disorder (BD) patients: it was found that, while PR did not closely correlate with symptomatology, some elements of PR differ between BD patients and patients with other severe mental illness (SMI). Moreover, as the role of caregiver, communication modalities within care and knowledge gained from peers were equally important, the authors suggested that these elements should be more broadly considered to improve recovery-focused care in BD. Subsequently, through a systematic review on 26 papers, Cheng and Lai identified the risk and protective factors for parental stress in families with children with special educational needs: as several risk and protective factors were found, it was suggested to design actions at social and mental health level to promote the wellbeing of parents at high risk of developing disabling mental conditions.

The topic of social support and quality of life was evaluated in chronically homeless patients (HP) with schizophrenia: Chen et al. provided results on 3.967 HP and 3.724 non-homeless patients (NHP) in rural China. As sociodemographic differences between groups emerged,the study established that HP, compared to NHP, showed lower scores at the *Social Support Rating Scale*, confirming that HP were unlikely to obtain social support. Furthermore, HP experienced more anxiety, depression, physical pain, reduced quality of life and were characterized by increased psychoticism and neuroticism, indicating also higher levels of antisocial behavior and emotional distress.

The role of social policies in facilitating access to mental health services is also addressed in Wu et al. who analyzed the effects a major natural disaster on the development of disabling mental disorders: the authors evaluated prevalence and symptoms of PTSD in a group of survivors after a tornado disaster: on 237 survivors, 13.6% of the sample was diagnosed with PTSD. Furthermore, being female and having suffered from severe property damage, increased the risk of developing PTSD. As nearly two-thirds of PTSD individuals did not seek mental assistance, due to stigma and lack of knowledge about psychological assistance, it was suggested that, in case of exposure to natural disasters, social policies should increase accessibility to mental health services through specific awareness campaigns.

Considering the role of Positive psychology in emphasizing positive qualities, Umucu et al. provided results from an exploratory study illustrating the difference in *character strengths* on 11.699 subjects presenting with different types of disabilities. They demonstrated a differential distribution of the *character strengths* construct, suggesting that the evaluation of *character strengths* in people with disabilities could improve the implementation of tailored rehabilitative interventions.

Then, the topic of work support was evaluated by Tan et al. providing preliminary results on the acceptability and effectiveness of the *Augmented Reality Games to Enhance Vocational Ability of Patients (REAP*) program in 15 adults with intellectual and developmental disabilities attending work therapy. REAP programs consisted of gamified augmented reality café training scenarios and bridging group activities to facilitate transfer of learning to the work context. Results indicated that REAP programs were useful for most of the participants and trainers, providing unique opportunities to acquire new skills. Subsequently, Hug et al. assessed work-related impairments in participation and the need for support among 93 adult patients in day-care/inpatient setting. They found that 76% of the patients expressed a need for support, of which 92% expressed interest in job coaching. However, about half of the sample received support from the treatment team, suggesting that the need for support was insufficiently met. As work and training were highly relevant topic for people with SMI, it was suggested that appropriate measures to support work and professional employment are urgently needed to promote recovery.

In a shared-decision making perspective, to better understand the non-adherence phenomenon in individuals with SMI, Asher et al. studied the complex processes of developing attitudes toward medication and decisional aspects on their patterns of use. They found that these processes progressed through distinct sequential phases that are dynamic and non-linear, suggesting that clinicians have to evaluate these processes through a shared dialogue with the patient focusing on personal attitudes and beliefs regarding medications. By doing this, therapeutic alliance could be further implemented in a recovery-oriented care model.

Concluding, as recovery, quality of life and well-being are actual milestones of Social Psychiatry and Psychiatric Rehabilitation, we believe that, strengthening the evidence covered in this Research Topic, will bring to improve the value of psychosocial and recovery-oriented actions in patients with SMI.

## Author contributions

AV: Writing – review & editing, Writing – original draft. JL: Writing – original draft.

